# Metastasis-Directed Therapy Versus Systemic Therapy in Oligometastatic Endometrial Carcinoma Recurrence: A Population-Based Registry Analysis

**DOI:** 10.3390/cancers18203382

**Published:** 2026-10-19

**Authors:** Paolo Gennari, Mandy Busse, Atanas Ignatov

**Affiliations:** Department of Obstetrics and Gynecology, Otto von Guericke University Magdeburg, 39120 Magdeburg, Germany; mandy.busse@med.ovgu.de (M.B.);

**Keywords:** endometrial carcinoma, oligometastatic recurrence, metastasis-directed therapy, stereotactic body radiotherapy, inverse probability of treatment weighting, propensity score, population-based registry, high-grade endometrial cancer

## Abstract

**Background/Objectives:** Comparative data on metastasis-directed therapy (MDT) versus systemic therapy in oligometastatic endometrial carcinoma (EC) recurrence are lacking. **Methods:** In this retrospective population-based registry study (Tumor Register Saxony-Anhalt, 2000–2025), we included 153 treated patients with distant-only oligometastatic EC recurrence (≤2 anatomical sites) after curative-intent primary treatment. Overall survival (OS) was compared between a strategy including MDT (*n* = 97) and systemic therapy alone (n = 56) using inverse probability of treatment weighting (IPTW)-adjusted Cox regression. Subgroup, sensitivity and competing risks analyses were exploratory. **Results:** In the IPTW analysis (n = 145 with complete data), OS did not differ significantly between MDT and systemic therapy (hazard ratio [HR] 0.90, 95% confidence interval [CI] 0.61–1.35; *p* = 0.622), consistent across sensitivity analyses. In exploratory subgroup analysis, MDT was associated with better OS in high-grade tumors (n = 51; HR 0.27, 95% CI 0.12–0.59; *p* = 0.001; Holm-adjusted *p* = 0.013), but not in low-grade tumors (HR 1.29; *p* = 0.339). **Conclusions:** MDT and systemic therapy showed no statistically significant survival difference in unselected oligometastatic EC recurrence; the confidence interval cannot exclude clinically relevant benefit or harm, supporting individualized decisions. The high-grade finding is hypothesis-generating and warrants prospective validation.

## Simple Summary

Endometrial cancer sometimes returns as a small number of distant metastases. Doctors can then treat these metastases directly, for example with surgery or focused radiotherapy, or treat the whole body with drugs such as chemotherapy. It is unknown which approach helps patients live longer. We analyzed data from a regional cancer registry in Germany covering 153 women whose cancer had returned in one or two distant sites. After statistical adjustment for differences between the groups, women treated directly at the metastases and women treated with drugs survived for a similar length of time. However, women with high-grade tumors appeared to live longer after direct treatment of the metastases. This finding is preliminary and needs to be confirmed in prospective studies before it can change clinical practice.

## 1. Introduction

Endometrial carcinoma (EC) is the most common gynecological malignancy in high-income countries and its incidence is rising globally, driven largely by increasing rates of obesity and an aging population [1]. In Europe, an estimated 121,578 new cases were diagnosed in 2018 [2]. The majority of patients present with early-stage disease and achieve favorable long-term outcomes following curative-intent surgery; the five-year relative survival of European women diagnosed with EC in 2000–2007 was 76% [2]. However, a clinically meaningful subset of patients develops recurrent disease, and in those with distant metastatic recurrence, prognosis remains poor, with median overall survival rarely exceeding two years [3,4]. In multicenter and population-based series, the pattern of recurrence and the feasibility of secondary radical surgery are major determinants of survival after recurrence, and five-year overall survival after non-vaginal recurrence in endometrioid EC was only 36.1%, versus 77.0% after isolated vaginal recurrence [4,5,6].

The concept of oligometastatic disease, first introduced by Hellman and Weichselbaum in 1995, describes an intermediate biological state between localized tumor and widespread systemic dissemination, characterized by a limited number of metastatic sites and a potentially restricted metastatic capacity [7]. In this conceptual framework, aggressive local treatment of metastatic deposits—termed metastasis-directed therapy (MDT)—may prevent progression to polymetastatic disease and improve survival, or even achieve long-term disease control in selected patients. This hypothesis has gained substantial clinical traction over the past decade, supported by the SABR-COMET randomized phase 2 trial, which demonstrated a significant improvement in overall survival with stereotactic ablative radiotherapy (SABR) added to standard systemic treatment in patients with one to five oligometastases across multiple tumor histologies [8].

In EC, oligometastatic recurrence occurs in a subgroup of patients with otherwise limited systemic tumor burden, most commonly involving pulmonary, hepatic, or lymph node sites [3,4]. The operational definition of oligometastatic disease in EC has not been standardized, but a framework based on the number of affected anatomical locations—rather than the absolute number of metastatic foci—has been proposed in head and neck squamous cell carcinoma, with a threshold of two or fewer sites identifying a population with meaningfully better prognosis [9]. Current European guidelines acknowledge the potential benefit of local ablative treatment in patients with oligometastatic EC recurrence but emphasize the lack of prospective evidence and the need for individualized multidisciplinary decision-making [2].

In clinical practice, patients with oligometastatic EC recurrence may be managed with MDT—encompassing surgical resection, stereotactic body radiotherapy (SBRT), or combinations thereof—or with systemic treatment, including chemotherapy, hormonal therapy, or immunotherapy-based regimens. Pulmonary metastasectomy in uterine malignancies has been associated with five-year overall survival rates of approximately 48–69% in selected series [10,11]. SBRT for gynecologic oligometastatic disease has similarly demonstrated high local control with acceptable toxicity in a systematic review and in pooled and single-center analyses [12,13,14]. Across tumor types, meta-analyses of randomized trials indicate that adding MDT to standard of care improves survival, although the effect varies by tumor type and clinical setting [15,16], and a randomized phase II trial supported the addition of MDT to systemic therapy in oligometastatic pancreatic cancer [17]. By contrast, non-randomized comparisons of local treatment versus systemic therapy alone have reported large survival advantages but are at serious risk of bias [18]. However, direct comparative data between MDT and systemic treatment in EC-specific oligometastatic cohorts are lacking.

The present study aimed to compare overall survival between MDT and systemic therapy in patients with oligometastatic EC recurrence using a large population-based registry dataset from Saxony-Anhalt, Germany. To account for the non-random treatment allocation inherent to observational data, we applied inverse probability of treatment weighting (IPTW) using the propensity score as the primary analytical approach [19]. Subgroup analyses were performed to identify patient subsets potentially deriving differential benefit from MDT.

## 2. Materials and Methods

### 2.1. Study Design and Data Source

We conducted a retrospective population-based cohort study using data from the Tumor Register Saxony-Anhalt (Tumorregister Sachsen-Anhalt), a mandatory regional cancer registry covering the federal state of Saxony-Anhalt, Germany. Data extraction covered the observation period from 2000 to 2025. The study was approved by the Ethics Committee of the Otto von Guericke University Magdeburg (approval no. 116/26) and conducted in accordance with applicable data protection regulations. The study is reported in accordance with the STROBE (Strengthening the Reporting of Observational Studies in Epidemiology) statement.

### 2.2. Patient Selection

**Figure 1:**
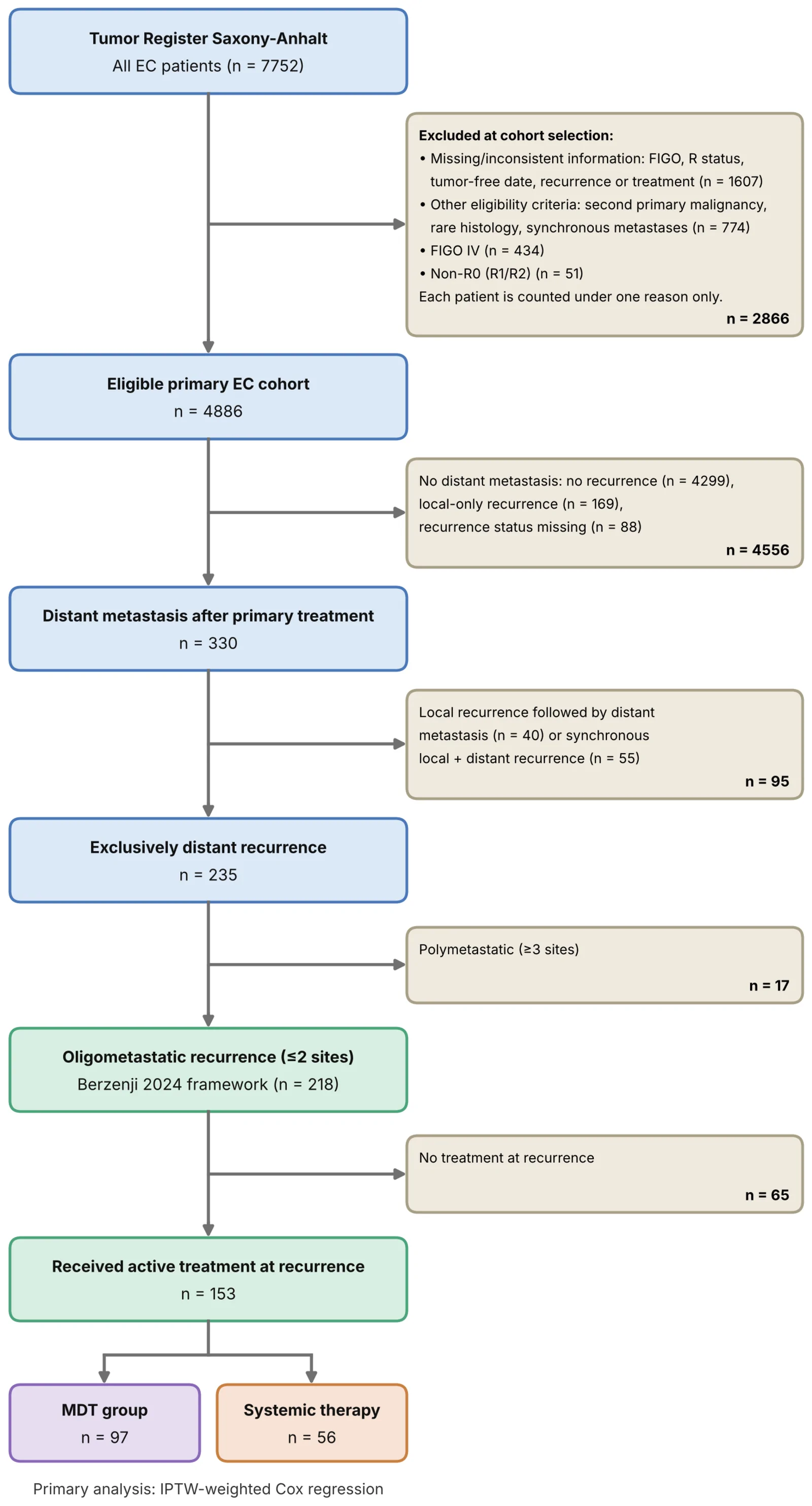
Study flowchart. Stepwise exclusion from the Tumor Register Saxony-Anhalt to the final analysis cohort. EC = endometrial carcinoma; FIGO = International Federation of Gynecology and Obstetrics; MDT = metastasis-directed therapy.

The study population was derived from a merged dataset of International Federation of Gynecology and Obstetrics (FIGO) stage I–II and selected FIGO stage III patients with EC registered in the Tumor Register Saxony-Anhalt. The stepwise exclusion process is summarized in Figure 1. Briefly, starting from 7752 registry patients, each excluded patient was assigned to a single reason at cohort selection: missing or inconsistent information on FIGO stage, resection status, tumor-free date, or recurrence and treatment data (n = 1607); other eligibility criteria, namely second primary malignancy, rare (mesenchymal) histology and synchronous or pre-treatment metastases (n = 774); FIGO stage IV disease (n = 434); and non-R0 resection (n = 51), leaving 4886 patients. The following exclusions were then applied: absence of any distant metastasis after primary treatment (n = 4556); local-only or mixed local-and-distant recurrence (n = 95); and polymetastatic recurrence involving three or more anatomical sites per the Berzenji 2024 [9] framework (n = 17). Of the 218 patients with distant-only oligometastatic recurrence (≤2 anatomical sites), 65 who received no treatment were excluded from survival comparisons, leaving 153 patients who received active treatment at recurrence for the primary analysis cohort.

### 2.3. Definition of Oligometastatic Disease

Oligometastatic recurrence was defined as distant metastatic recurrence involving a maximum of two distinct anatomical sites, consistent with the location-based framework proposed by Berzenji et al. [9]. Individual metastatic sites recorded in the registry included pulmonary, hepatic, lymph nodal, peritoneal, osseous, cerebral, cutaneous, pleural, adrenal and other-organ locations (Table 1). Recurrences at the vaginal vault were classified as local recurrences and excluded, whereas vaginal lesions registered as metastases (non-vault) were counted as distant, in accordance with the registry coding of recurrence type. The effect of this classification on the results was examined in a sensitivity analysis (SA7).

### 2.4. Treatment Classification

Treatment received for the index oligometastatic recurrence was classified into two groups based on treatment modality, irrespective of a fixed time window; all treated patients were included regardless of the interval between recurrence and treatment initiation. Because survival was measured from the first distant metastasis, this interval constitutes potential immortal time [20,21]; it was evaluated by comparing time to treatment between groups and by sensitivity analyses (SA) SA2, SA3 and SA6. The MDT group (n = 97) therefore represents a treatment strategy that includes MDT rather than MDT alone; it included patients treated with surgery alone, radiotherapy alone, combined surgery and radiotherapy, or MDT combined with systemic therapy (n = 19). The systemic therapy group (n = 56) included patients treated with chemotherapy alone, hormonal therapy alone, immunotherapy or targeted therapy, or combinations thereof. Accordingly, the primary comparison is between a strategy including MDT and systemic therapy alone; the comparison of pure MDT with systemic therapy was addressed in SA1. Three sensitivity analyses tested the robustness of the primary classification: a pure MDT definition excluding concurrent systemic therapy (SA1), and two exploratory analyses excluding patients whose treatment began more than three (SA2) or six (SA3) months after the index recurrence. Two further post hoc analyses addressed confounding by metastatic site (SA4 and SA5; Section 2.7). A further post hoc analysis (SA6) re-set time zero to the start of treatment, which removes the interval between recurrence and treatment from the observation time. Three further post hoc analyses (SA7–SA9; Section 2.7) addressed the classification of vaginal and pelvic lesions, the exclusion of cerebral and other-organ metastases, and treatment era.

### 2.5. Outcome Measures

The primary outcome was overall survival (OS) following first distant metastatic recurrence, defined as the time from the date of first documented distant metastasis to death from any cause or censoring at the last registry contact date. For the competing risks analysis, events were classified as tumor-related death (event type 1), non-tumor-related death (event type 2), or censoring (event type 0).

### 2.6. Covariates

The following baseline covariates were included in the propensity score model: age at first distant metastasis (continuous), FIGO stage (binary: I–II vs. III), histological type (binary: endometrioid vs. non-endometrioid), histological grade (binary: low vs. high grade; high grade defined as FIGO grade 3 of any histology; serous, clear cell and carcinosarcoma histologies were classified as high grade unless a low grade was documented, n = 1), adjuvant therapy (binary: yes vs. no), number of metastatic sites (1 vs. 2), and time to recurrence (binary: ≤12 vs. >12 months). Covariate completeness was 100% for all variables except histological grade (145/153, 94.8%). Metastatic sites were not included in the primary propensity score model because of sparse categories and limited overlap (cerebral involvement, n = 10, all in the MDT group; other-organ sites, 28 of 30 in the MDT group); they were addressed in sensitivity analyses SA4, SA5, SA7 and SA8. Consequently, IPTW balanced only the seven covariates included in the propensity score model; balance on metastatic sites was not achieved (Table 1).

### 2.7. Statistical Analysis

Baseline characteristics were compared using chi-squared or Fisher’s exact tests and the Mann–Whitney U test. A logistic regression model estimated the propensity score (probability of receiving MDT). Stabilized IPTW weights were trimmed at the 99th percentile. Patients with missing histological grade (n = 8) could not be included in the propensity score model; weighted analyses therefore comprised 145 of the 153 treated patients. Covariate balance was assessed using standardized mean differences (SMD < 0.10 = adequate balance). The primary analysis used an IPTW-weighted Cox model; the secondary analysis used an unweighted multivariable Cox model. Exploratory subgroup analyses (twelve subgroups defined by six baseline characteristics: FIGO stage, histological type, histological grade, number of metastatic sites, time to recurrence and pulmonary involvement at first recurrence) were performed using unweighted multivariable Cox models adjusted for the remaining covariates and are interpreted as hypothesis-generating. Because these analyses were exploratory and not pre-specified, *p*-values are reported both unadjusted and adjusted for multiplicity using the Holm procedure, applied separately to the twelve subgroup estimates and to the six interaction tests (Bonferroni-adjusted values are given in Table S3, Panels A and B). Effect modification was formally assessed for each of the six characteristics by adding an MDT × subgroup-variable product term to the unweighted multivariable Cox model (Wald test; the grade interaction was also tested in the IPTW-weighted model and after exclusion of other-organ sites). Sensitivity analyses included SA1 (pure MDT), SA2 (exclusion of treatment initiated >3 months after the index recurrence), and SA3 (exclusion of treatment initiated >6 months after the index recurrence). Two further post hoc sensitivity analyses, added after review of the baseline imbalance in metastatic sites, addressed confounding by metastatic site: SA4, additional adjustment of the propensity score for pulmonary and hepatic involvement at first recurrence, and SA5, exclusion of patients with other-organ metastatic sites (n = 30). A sixth post hoc sensitivity analysis, SA6, addressed immortal time directly by measuring survival from the start of treatment instead of from the first distant metastasis (one patient who died before treatment start was excluded). Three further post hoc sensitivity analyses were performed: SA7, exclusion of patients with vaginal (n = 8) or pelvic (n = 2) lesions registered as distant metastases; SA8, exclusion of patients with cerebral (n = 10) or other-organ (n = 30) metastases, i.e., restriction to metastatic sites represented in both treatment groups; and SA9, additional adjustment of the propensity score for the calendar year of first distant metastasis (continuous) to address treatment-era effects. Competing risks analyses used cause-specific Cox models and Aalen-Johansen estimators. The proportional hazards assumption was assessed with scaled Schoenfeld residuals, and median follow-up was estimated with the reverse Kaplan–Meier method. All analyses were performed using IBM SPSS Statistics v29 (IBM Corp., Armonk, NY, USA) and R v4.3.3 (R Foundation for Statistical Computing, Vienna, Austria).

## 3. Results

### 3.1. Study Population and Baseline Characteristics

Of 7752 patients with EC registered in the Tumor Register Saxony-Anhalt between 2000 and 2025, 218 met all inclusion criteria for distant-only oligometastatic recurrence following curative-intent primary treatment (Figure 1). Of these, 153 patients (70.2%) received active treatment and formed the primary analysis cohort; 65 patients (29.8%) received no treatment and were excluded from survival comparisons. Among the 153 treated patients, 97 (63.4%) received MDT and 56 (36.6%) received systemic therapy.

Baseline characteristics of the treated cohort are summarized in Table 1. The two treatment groups were comparable with respect to age (median 69 vs. 71 years; *p* = 0.567), FIGO stage III disease (35.1% vs. 42.9%; *p* = 0.432), histological type (*p* = 0.650), grade (*p* = 0.757), adjuvant therapy receipt (*p* = 0.145; the distribution of adjuvant modalities differed, *p* = 0.020, with chemotherapy alone more frequent in the systemic therapy group), number of metastatic sites (*p* = 0.375), and time to first distant recurrence (median 24.0 vs. 24.0 months; *p* = 0.180). Significant differences remained in the distribution of metastatic sites at first recurrence: pulmonary (26.8% vs. 55.4%; *p* < 0.001) and hepatic (4.1% vs. 19.6%; *p* = 0.005) metastases were less frequent, and cerebral (10.3% vs. 0%; *p* = 0.014) and other-organ sites (28.9% vs. 3.6%; *p* < 0.001) more frequent, in the MDT group, reflecting confounding by indication. Other-organ sites (vaginal, n = 8; soft tissue or abdominal wall, n = 8; intestinal, n = 5; urinary tract, n = 3; pelvic, n = 2; unspecified, n = 5), all registered as distant recurrences, were concentrated in the MDT group (28 of 30 patients). Median time from first distant metastasis to treatment initiation was 10 days in the MDT group and 25 days in the systemic therapy group (*p* < 0.001).

### 3.2. Unadjusted Survival Analysis

**Figure 2:**
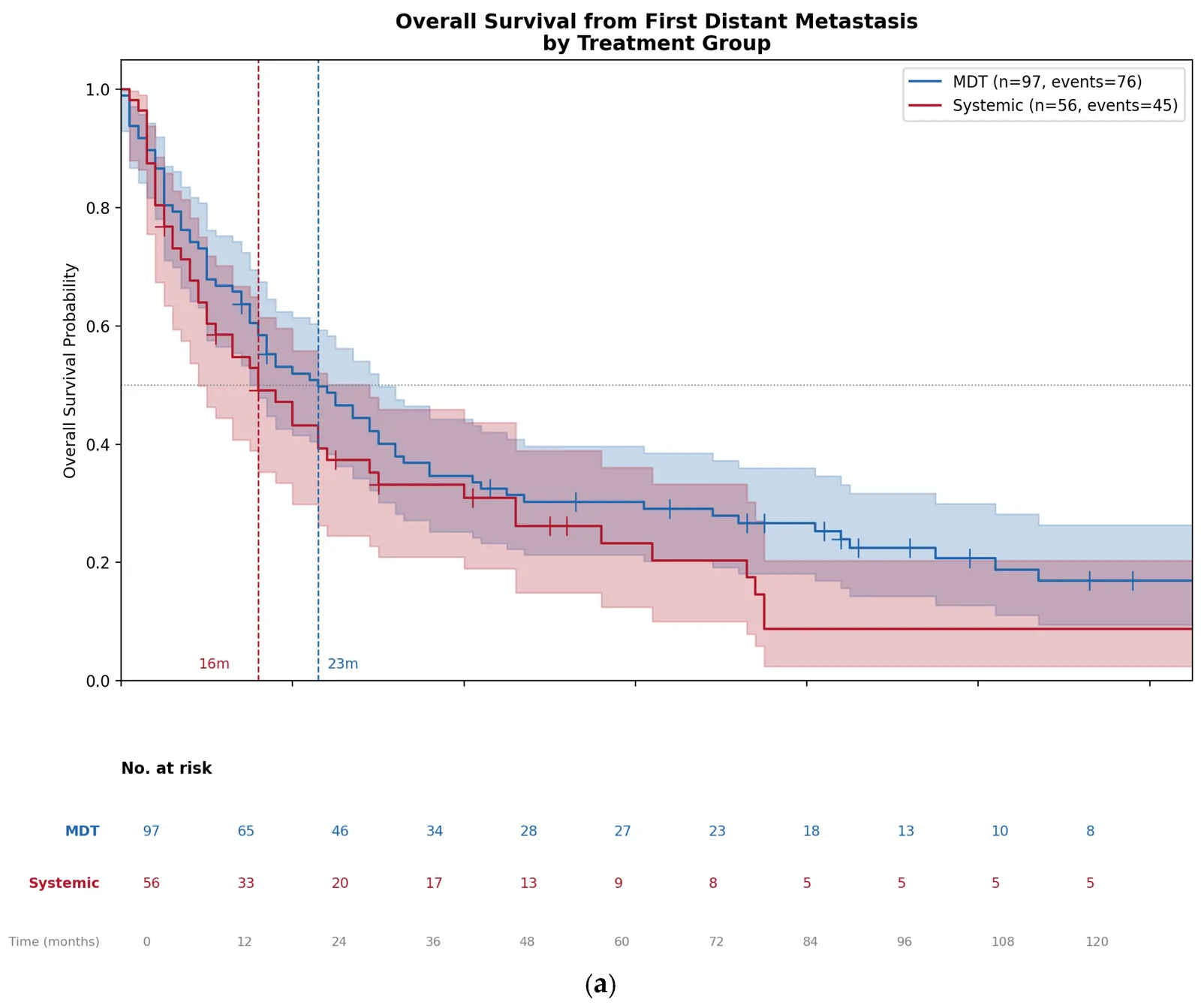

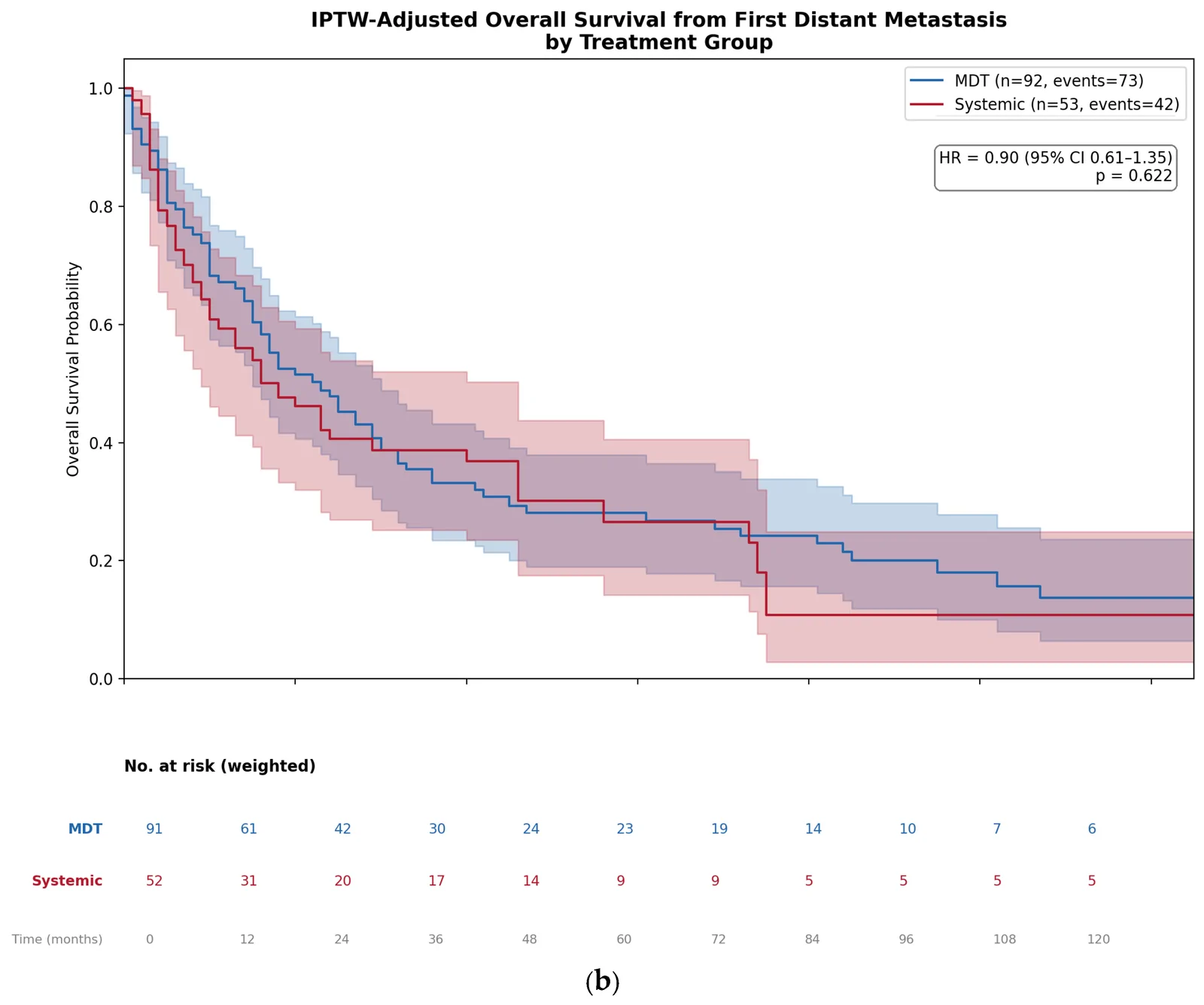
(**a**). Unadjusted Kaplan–Meier overall survival curves from first distant metastasis by treatment group. Blue: MDT (n = 97, events = 76); Red: systemic therapy (n = 56, events = 45). Shaded areas represent 95% confidence intervals. The dotted horizontal line indicates 50% survival probability. Dashed vertical lines indicate median overall survival (MDT: 23 months; systemic: 16 months). Log-rank *p* = 0.202. Number at risk shown below the *x*-axis. The *x*-axis is truncated at 120 months for display. (**b**). IPTW-adjusted Kaplan–Meier overall survival curves from first distant metastasis by treatment group. Blue: MDT (n = 92, events = 73); Red: systemic therapy (n = 53, events = 42). Stabilized IPTW weights trimmed at the 99th percentile; effective sample size: 140.5. HR = 0.90 (95% CI 0.61–1.35); *p* = 0.622. The *x*-axis is truncated at 120 months for display.

Median follow-up was 113 months (95% CI 86–146). In unadjusted Kaplan–Meier analysis, median OS from first distant metastasis was 23.0 months in the MDT group and 16.0 months in the systemic therapy group. Estimated OS rates at 12, 24, and 60 months were 66.8%, 48.7%, and 30.2% in the MDT group and 58.5%, 37.3%, and 23.3% in the systemic therapy group, respectively (log-rank *p* = 0.202). Unadjusted Kaplan–Meier curves are presented in Figure 2a.

### 3.3. Propensity Score Model and IPTW Balance

After application of stabilized IPTW weights trimmed at the 99th percentile, all seven covariates achieved SMD < 0.10, indicating adequate balance for these covariates (Figure S1). The effective sample size after weighting was 140.5. Balance was not achieved for metastatic sites, which were not included in the propensity score model (Table 1).

### 3.4. Primary Analysis: IPTW-Weighted Cox Model

In the primary IPTW-weighted Cox model (n = 145 with complete data: 92 MDT, 53 systemic therapy), a strategy including MDT was not associated with a statistically significant difference in OS compared with systemic therapy alone (HR 0.90, 95% CI 0.61–1.35; *p* = 0.622); the confidence interval cannot exclude either a clinically relevant benefit or harm of MDT. The IPTW-adjusted Kaplan–Meier curves are presented in Figure 2b.

### 3.5. Secondary Analysis: Multivariable Cox Model

In the unweighted multivariable Cox model, the HR for MDT was 0.77 (95% CI 0.52–1.16; *p* = 0.217). Age at metastasis was independently associated with OS (HR per year 1.03; *p* = 0.026), and high histological grade was associated with significantly worse survival (HR 2.20, 95% CI 1.40–3.47; *p* = 0.001). The proportional hazards assumption was not violated for MDT (*p* = 0.75); the global test was significant (*p* = 0.014), driven by histological grade (*p* = 0.048). Full results are reported in Table 2.

### 3.6. Subgroup Analyses

**Figure 3:**
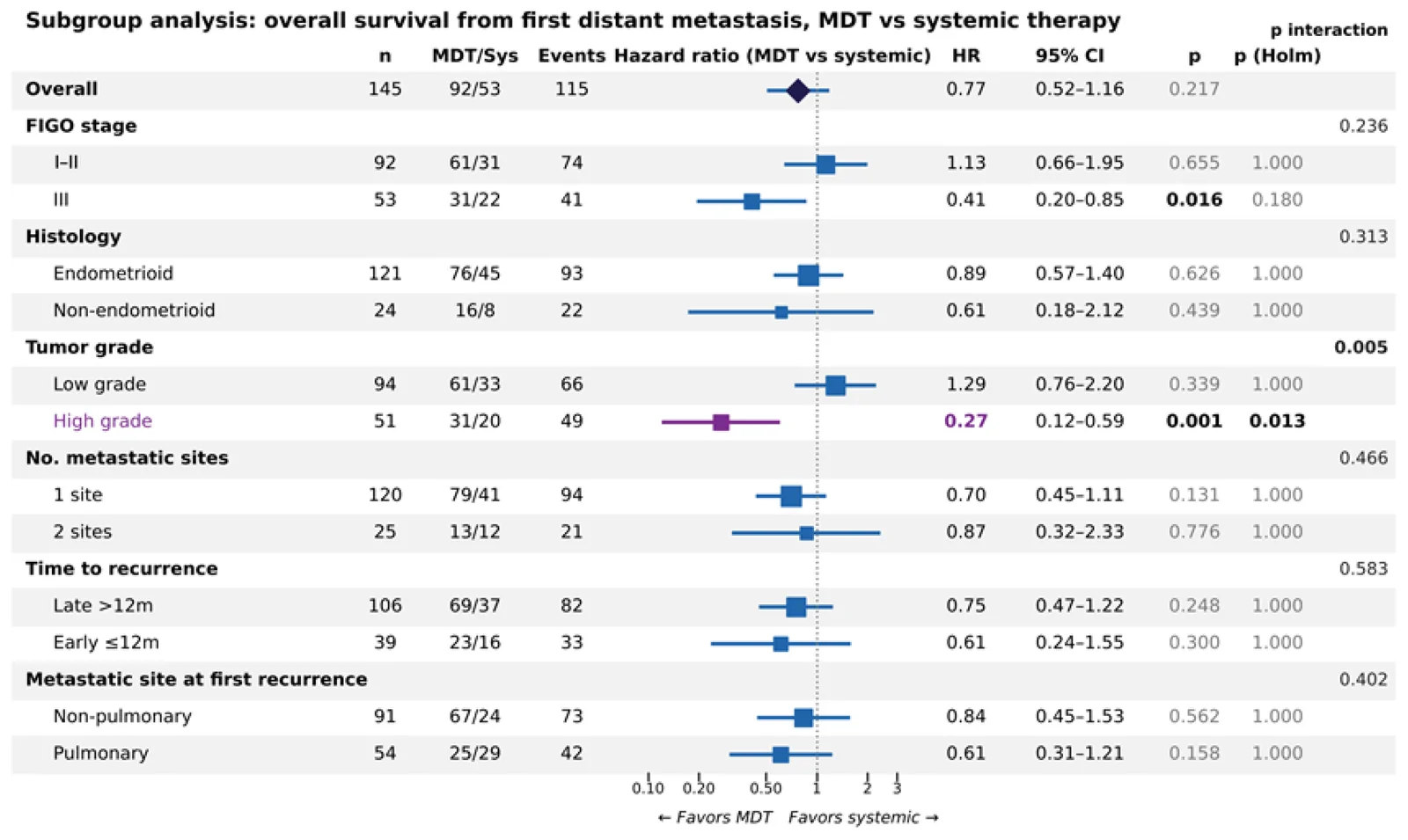
Exploratory subgroup forest plot for overall survival (MDT vs. systemic therapy). Hazard ratios and 95% confidence intervals from unweighted multivariable Cox models within each subgroup. n = number of patients; MDT/Sys = number of patients in each treatment group; Events = number of deaths. The diamond indicates the overall estimate; squares indicate subgroup estimates (size proportional to subgroup sample size); the dotted vertical line marks HR = 1. *p* = unadjusted *p*-value; *p* (Holm) = *p*-value adjusted for the twelve subgroup estimates using the Holm procedure; *p* interaction = unadjusted *p*-value of the MDT × subgroup-variable interaction test (Holm-adjusted *p* for tumor grade: 0.031). Bold *p*-values indicate *p* < 0.05; the high-grade subgroup is highlighted in purple. The overall estimate shown is from the unweighted multivariable Cox model; the primary IPTW estimate is HR 0.90 (95% CI 0.61–1.35). Metastatic site refers to the first distant recurrence. All subgroup analyses are exploratory and hypothesis-generating. CI = confidence interval; HR = hazard ratio; MDT = metastasis-directed therapy.

Exploratory subgroup analyses are presented in Figure 3 and Table S3 (Panel A). In the overall cohort, the HR for MDT was 0.77 (95% CI 0.52–1.16; *p* = 0.217). A nominally significant benefit of MDT was identified in the high-grade subgroup (HR 0.27, 95% CI 0.12–0.59; *p* = 0.001; Holm-adjusted *p* = 0.013), representing a 73% lower hazard of death, and in the FIGO III subgroup (HR 0.41, 95% CI 0.20–0.85; *p* = 0.016; Holm-adjusted *p* = 0.18). Neither benefit was observed in the corresponding low-grade (HR 1.29; *p* = 0.339) or FIGO I–II (HR 1.13; *p* = 0.655) subgroups. After Holm adjustment for the twelve subgroup estimates, only the high-grade estimate remained statistically significant. The non-endometrioid subgroup showed a directionally consistent but non-significant MDT benefit (HR 0.61; *p* = 0.439). After exclusion of patients with other-organ sites, the high-grade HR was 0.37 (95% CI 0.16–0.86; *p* = 0.022) and the FIGO III HR was 0.50 (95% CI 0.21–1.15; *p* = 0.103). Formal interaction tests between MDT and each subgroup variable (Table S3, Panel B) were statistically significant only for tumor grade (*p* = 0.005; Holm-adjusted *p* = 0.031); they were not significant for FIGO stage III (*p* = 0.24), non-endometrioid histology (*p* = 0.31), number of metastatic sites (*p* = 0.47), early recurrence (*p* = 0.58) or pulmonary involvement at first recurrence (*p* = 0.40). The grade interaction remained nominally significant in the IPTW-weighted model (*p* = 0.001) and after exclusion of other-organ sites (*p* = 0.040, unadjusted).

### 3.7. Sensitivity Analyses

All nine sensitivity analyses were consistent with the primary result of no significant difference (Table 3; Table S1; Figure S2). SA1 (pure MDT, n = 129): HR 0.83 (95% CI 0.55–1.23; *p* = 0.351). This analysis compares MDT alone with systemic therapy alone and gave a result consistent with the primary analysis. SA2 (exclusion of treatment >3 months after recurrence, n = 131): HR 0.89 (95% CI 0.58–1.35; *p* = 0.569). SA3 (exclusion of treatment >6 months after recurrence, n = 140): HR 0.89 (95% CI 0.60–1.33; *p* = 0.570). SA4 (additional adjustment for pulmonary and hepatic involvement, n = 145): HR 0.84 (95% CI 0.54–1.33; *p* = 0.468). SA5 (exclusion of the 30 patients with other-organ sites, 28 of whom received MDT; n = 115): HR 1.04 (95% CI 0.69–1.58; *p* = 0.845), i.e., a point estimate close to unity. SA6 (survival measured from the start of treatment, n = 144): HR 0.91 (95% CI 0.61–1.36; *p* = 0.636), essentially identical to the primary analysis. Because the interval between recurrence and treatment is removed from the observation time in this analysis, it directly addresses immortal-time bias. SA7 (exclusion of the ten patients with vaginal or pelvic lesions registered as distant metastases, n = 135): HR 0.94 (95% CI 0.63–1.41; *p* = 0.772). SA8 (exclusion of the 40 patients with cerebral or other-organ metastases, n = 105): HR 0.89 (95% CI 0.57–1.39; *p* = 0.609). SA9 (additional adjustment of the propensity score for the calendar year of first distant metastasis, n = 145): HR 0.90 (95% CI 0.60–1.33; *p* = 0.587). Cerebral involvement (n = 10, all in the MDT group) could not be included in the propensity score because of non-overlap. Sample sizes refer to patients contributing to each weighted model, i.e., after applying the respective restriction to the 153 treated patients (SA1: 19 patients treated with MDT plus systemic therapy excluded; SA2: 14 and SA3: 5 patients whose treatment began more than 3 or 6 months after the index recurrence excluded; SA7: 10 and SA8: 40 patients with the specified metastatic sites excluded) and after exclusion of the 8 patients with missing histological grade.

### 3.8. Competing Risks Analysis

**Figure 4:**
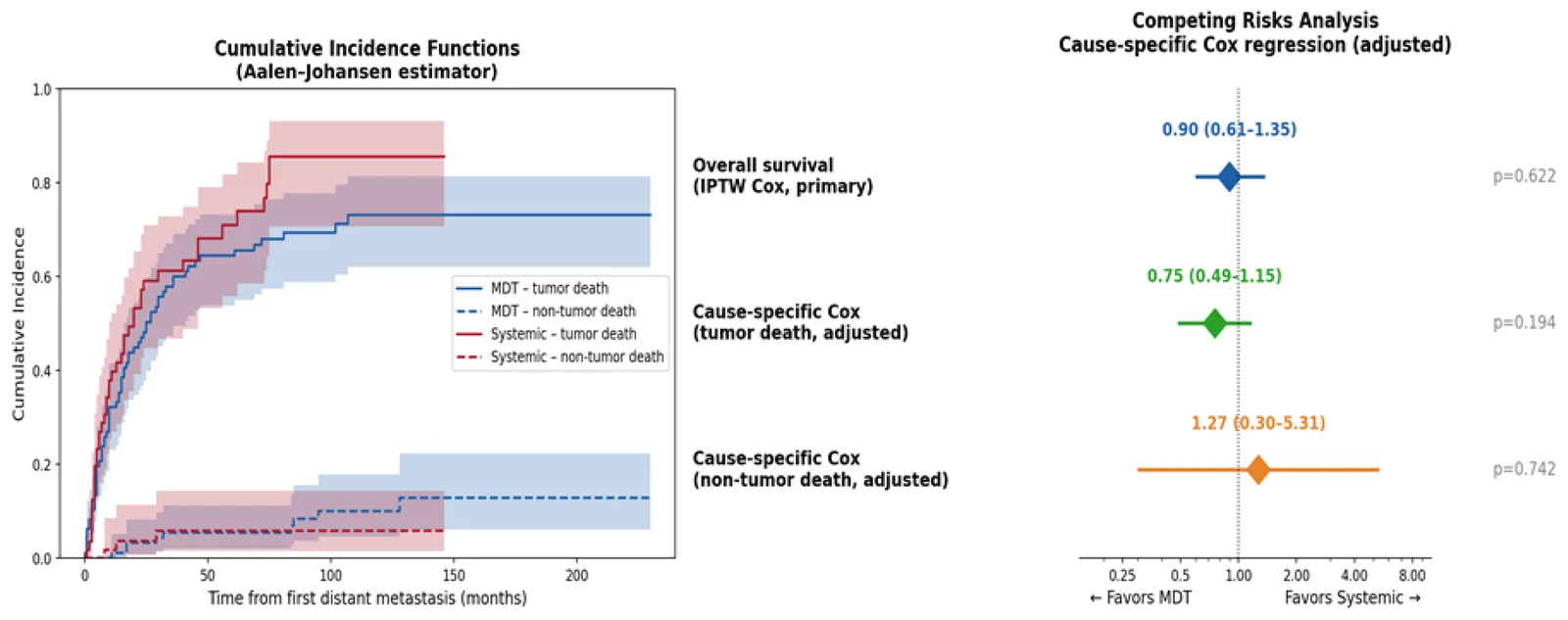
Competing risks analysis. **Left**: Cumulative incidence functions (Aalen-Johansen estimator; all 153 treated patients) for tumor-related death (solid lines) and non-tumor-related death (dashed lines) by treatment group (MDT: blue; systemic: red). Shaded areas represent 95% confidence intervals. **Right**: Cause-specific Cox regression hazard ratios for overall survival (IPTW primary), tumor-related death, and non-tumor-related death, the latter two adjusted for age, FIGO stage, histology, grade, adjuvant therapy, number of metastatic sites, and time to recurrence (N = 145). Horizontal bars indicate 95% CIs; the dotted vertical line marks HR = 1. Wide CI for non-tumor death reflects low event count (n = 12). CIF = cumulative incidence function; HR = hazard ratio; MDT = metastasis-directed therapy.

Among 153 treated patients, 109 (71.2%) died of tumor-related causes, 12 (7.8%) of non-tumor causes, and 32 (20.9%) were censored. Adjusted cause-specific Cox analysis (N = 145 after exclusion of the 8 patients with missing grade, comprising 103 tumor-related and 12 non-tumor deaths) gave an HR for tumor-related death of 0.75 (95% CI 0.49–1.15; *p* = 0.194) and for non-tumor death of 1.27 (95% CI 0.30–5.31; *p* = 0.742). Results are presented in Figure 4 and Table S2.

## 4. Discussion

The present population-based registry study examined overall survival outcomes in patients with oligometastatic EC recurrence treated with a strategy including MDT versus systemic therapy alone. In the primary IPTW-weighted analysis, no statistically significant difference in OS was observed, a finding consistent across the sensitivity analyses and the exploratory competing risks analysis. The most notable exploratory finding emerged from subgroup analysis: patients with high-grade tumors showed an apparent OS benefit associated with MDT, which remained statistically significant after correction for multiplicity.

No statistically demonstrable difference at the population level: clinical meaning and context. The absence of a statistically significant OS difference between MDT and systemic therapy should not be interpreted as evidence of MDT inferiority, nor as evidence of equivalence: the 95% confidence interval (0.61–1.35) is compatible with both a clinically relevant benefit and a clinically relevant harm of MDT, and this study was not designed or powered to establish equivalence. What can be said is that no statistically demonstrable population-level difference was found, supporting individualized treatment selection pending better-powered evidence. This interpretation is consistent with the limited existing literature. Rütten et al. recently reviewed the local and systemic treatment options for recurrent EC [3]. In a large Italian multicenter series, the pattern of recurrence and the feasibility of secondary radical surgery, but not histotype or grade, were independent predictors of post-relapse survival [4], and in a molecularly classified cohort the molecular subgroup was a significant indicator of survival after recurrence [5]. Current guidelines of the European Society of Gynaecological Oncology, the European Society for Radiotherapy and Oncology and the European Society of Pathology (ESGO/ESTRO/ESP) acknowledge MDT as a potentially beneficial option but stop short of a firm recommendation due to the absence of prospective comparative evidence [2]. Point estimates across the primary, secondary and sensitivity analyses mostly favored MDT (HR range 0.77–0.94), with the exception of the analysis excluding other-organ sites (HR 1.04); all confidence intervals included unity, so a modest population-level benefit can neither be demonstrated nor excluded in this study. It should also be noted that the MDT group comprised surgery, radiotherapy, their combination and MDT combined with systemic therapy (n = 19); the comparison made here is therefore between a treatment strategy that includes MDT and systemic therapy alone, not between MDT as a single modality and systemic therapy. The analysis restricted to pure MDT (SA1: HR 0.83, 95% CI 0.55–1.23) gave a consistent result.

The high-grade subgroup: a hypothesis-generating signal. The 73% reduction in the hazard of death in the high-grade subgroup was not observed in the low-grade subgroup, suggesting possible effect modification by tumor grade. A formal interaction test supported effect modification by grade (Holm-adjusted *p* = 0.031), whereas the interaction with FIGO stage was not significant (*p* = 0.24). Of the twelve subgroup estimates, only the high-grade result remained statistically significant after Holm adjustment (adjusted *p* = 0.013). Because grade was not a pre-specified effect modifier and the high-grade subgroup comprised only 51 patients, the magnitude of this effect is likely to be overestimated. A nominally significant MDT benefit was also observed in the FIGO III subgroup, which is biologically enriched for high-grade disease, but did not remain significant after multiplicity adjustment (adjusted *p* = 0.18), whereas the non-endometrioid subgroup showed only a directionally consistent, non-significant benefit. High-grade EC—encompassing grade 3 endometrioid carcinoma, uterine serous carcinoma, and clear cell carcinoma—is characterized by aggressive tumor biology [22,23]; in the PORTEC-3 trial of high-risk EC, distant metastases were the most frequent first site of relapse [24], and in a molecularly classified cohort of recurrent EC, p53-abnormal tumors had the shortest survival after recurrence (median 10 months) [5]. If the oligometastatic state in high-grade EC represents a biologically early phase of systemic spread, effective local eradication may interrupt the metastatic cascade more decisively than systemic therapy alone, consistent with the oligometastatic hypothesis of Hellman and Weichselbaum [7]. Both subgroup signals should nevertheless be interpreted cautiously: after exclusion of patients with other-organ sites the high-grade estimate was attenuated but remained significant (HR 0.37, 95% CI 0.16–0.86; *p* = 0.022), whereas the FIGO III estimate was no longer significant (HR 0.50, 95% CI 0.21–1.15; *p* = 0.103).

MDT in gynecologic oncology: existing evidence. The SABR-COMET trial demonstrated a significant OS benefit of SABR added to standard of care across multiple tumor histologies [8], but was not designed to address gynecologic primaries. More directly relevant is the multi-institutional analysis by Donovan et al. (215 patients, of whom 107 had endometrial primaries), which reported five-year OS of 33.1% and a five-year cumulative incidence of local recurrence of 18.5% [12]; a systematic review of 667 patients with oligometastatic gynecologic malignancies likewise reported local control rates of 71–100%, but also a high rate of out-of-field progression [14]. Pulmonary metastasectomy series in uterine malignancies report five-year OS of 48–69% following complete resection [10,11]. In a series restricted to oligometastatic uterine cancer, Reddy et al. reported local control in 80.3% of lesions without grade 3–4 toxicity [13]. These series are uncontrolled, however, and cannot separate the effect of MDT from patient selection. Outside gynecologic oncology, meta-analyses of randomized trials show improved survival with MDT overall, but not in breast cancer or oligoprogressive disease [15], and with an overall survival benefit in recurrent but not de novo oligometastatic disease [16]; in oligometastatic prostate cancer, the pooled STOMP and ORIOLE trials showed a sustained progression-free survival benefit of MDT over observation [25].

Systemic therapy: evolving standards. The systemic therapy comparator reflects real-world practice over a 25-year period, in which chemotherapy alone was the most frequent modality (71.4% of the systemic group); in a European real-world series of recurrent or advanced EC, median OS with first-line systemic therapy was 20.7 months [26]. Carboplatin and paclitaxel (GOG-209) is the established first-line chemotherapy backbone in advanced or recurrent EC [27], although the registry does not record individual regimens. The therapeutic landscape has evolved substantially with lenvatinib plus pembrolizumab (KEYNOTE-775), which demonstrated significant OS improvements in previously treated advanced EC [28]. These advances may further shift the risk-benefit calculus in contemporary practice.

Multimodal management of metastatic gynecologic malignancies. The integration of local ablative treatment into multimodal care has also been explored in other gynecologic primaries, where the evidence is likewise confined to uncontrolled series. In oligometastatic ovarian cancer, a recent systematic review and meta-analysis of eight studies including 594 patients reported one-year local control rates of 86.7–94.4% after SBRT, grade ≥ 3 toxicity in 0–6.1% of patients and no treatment-related deaths; SBRT was frequently delivered during treatment-free intervals or to control oligoprogression under maintenance with poly(ADP-ribose) polymerase (PARP) inhibitors, i.e., as a complement to, rather than a replacement for, systemic therapy [29]. A narrative review of SBRT in gynecologic oligometastases similarly concluded that outcomes and toxicity are encouraging, but that SBRT remains underutilized in the absence of randomized trials [30]. In cervical cancer, a single-center series of 56 patients with one to three extracranial oligometastases treated with definitive radiotherapy, surgery or ablation reported 3-year overall survival of 40.1% for supraclavicular and 55.2% for non-supraclavicular lesions, with long-term survival particularly in patients with small (≤15 mm) lesions [31]. In a cohort of 139 patients with cervical cancer treated with primary chemoradiotherapy, oligometastatic recurrence was associated with better progression-free and disease-specific survival after recurrence irrespective of the type of recurrence therapy, and in an exploratory comparison, surgical resection with or without adjuvant therapy (n = 12) was associated with improved progression-free survival after recurrence compared with palliative systemic treatment (n = 7) [32]. Single case reports illustrate both the potential of a multimodal approach in selected patients, such as a complete response with prolonged survival in a young woman with oligometastatic cervical adenosquamous carcinoma [33], and the limits of surgery in rapidly progressive extragenital disease, as in a case of uterine metastasis from small-cell lung cancer in which hysterectomy served symptom palliation only [34]. These reports share features that are also relevant to our study: patients were selected by lesion number, size and site; local treatment was frequently combined with systemic therapy; and comparative data are lacking. They support the view that MDT is best regarded as one component of multimodal, multidisciplinary management of selected patients rather than as an alternative to systemic therapy, which is how the primary comparison of the present study was framed, and they underline the need for prospective studies defining the patients most likely to benefit.

Limitations. As in any real-world registry analysis, several limitations apply. First, the retrospective design and the 25-year observation period (2000–2025) introduce treatment era effects: diagnostic work-up, surgical and radiotherapeutic techniques and, in particular, systemic regimens changed substantially over this period, and the registry does not record individual regimens. MDT use varied between 55% and 77% across four calendar periods (2000–2009, 2010–2014, 2015–2019 and 2020–2025), and additional adjustment of the propensity score for calendar year did not change the estimate (SA9: HR 0.90, 95% CI 0.60–1.33), but residual era effects, including the evolution of the systemic comparator, cannot be excluded. Classification of treatment into two broad categories conceals within-group heterogeneity; SA1, restricted to pure MDT, showed a consistent result. Second, the subgroup analyses were exploratory, not pre-specified and based on small samples (the high-grade subgroup involved 51 patients); although Holm-adjusted *p*-values are reported, adjustment cannot remove the risk of false-positive findings and of overestimated effect sizes inherent to post hoc subgroup analyses, and replication in independent cohorts is necessary. Exclusion of patients with mixed local and distant recurrence (n = 95) limits generalizability to the broader EC recurrence population. Eastern Cooperative Oncology Group (ECOG) performance status is not systematically captured in the registry and could therefore not be included in the propensity score model. Third, IPTW adjustment can only balance the measured covariates included in the propensity score model, so that unmeasured confounding remains possible, and residual confounding by indication—including on unmeasured functional status—cannot be excluded, as reflected in the significant between-group differences in pulmonary, hepatic, cerebral and other-organ metastatic sites. In particular, other-organ sites (heterogeneous: vaginal, soft-tissue or abdominal-wall, intestinal, urinary-tract and pelvic sites, all registered as distant recurrences) were concentrated in the MDT group (28 of 30 patients) and their prognostic significance is uncertain; excluding these patients moved the point estimate to unity (SA5), and cerebral involvement (all 10 patients in the MDT group) could not be balanced by weighting. Restriction to metastatic sites represented in both treatment groups (SA8: exclusion of cerebral and other-organ sites) gave an estimate close to the primary analysis (HR 0.89, 95% CI 0.57–1.39), as did exclusion of vaginal and pelvic lesions, which may be regarded as locoregional recurrences in some settings (SA7: HR 0.94, 95% CI 0.63–1.41); these analyses are, however, based on smaller samples (n = 135 and n = 105). As with most registry-based analyses, the completeness of recurrence event capture and subsequent treatment documentation may be lower than that of primary diagnosis data, and unrecorded recurrences or treatments cannot be excluded. Molecular classification (POLE, mismatch repair, p53), which is prognostic in recurrent EC [5], is not recorded in the registry and could not be considered. The definition of oligometastatic disease used here (two or fewer anatomical sites, with lymph nodes counted as a site) derives from a framework developed in another tumor type [9] and differs from definitions used elsewhere. In addition, because the 65 untreated patients were excluded, the analysis is conditional on receipt of treatment, which may select patients who survived long enough to be treated, although time to treatment was short in both groups. Finally, treatment was classified by modality without a fixed time window between recurrence and treatment initiation, so that the interval between recurrence and treatment constitutes potential immortal time. Time to treatment was in fact shorter for MDT than for systemic therapy (median 10 vs. 25 days; *p* < 0.001), which would bias the comparison against MDT rather than in its favor, and the result was unchanged when late-treated patients were excluded (SA2, SA3) or when survival was measured from the start of treatment (SA6, HR 0.91). Residual susceptibility bias related to why some patients were treated later than others cannot, however, be fully excluded. Time-dependent exposure modeling, landmark analyses and target-trial emulation have been proposed to address immortal time more formally [35,36,37] and should be considered in future analyses.

Clinical implications and future directions. Randomized trials or prospective registries in oligometastatic EC recurrence should incorporate grade-stratified analyses as a pre-specified objective. The HR 0.27 in the high-grade subgroup provides a rationale for grade-stratified confirmatory analyses, although an estimate from an exploratory subgroup is likely to be inflated. International multicenter collaboration is essential given the rarity of the oligometastatic EC phenotype, and molecular subtyping (POLE, mismatch repair, p53) should be incorporated into future study designs.

## 5. Conclusions

In this population-based registry analysis, no statistically significant difference in overall survival was observed between a treatment strategy including metastasis-directed therapy and systemic therapy alone in the unselected treated population; the confidence interval cannot exclude either a clinically relevant benefit or harm of MDT, supporting individualized treatment decisions guided by multidisciplinary tumor board assessment. A statistically significant OS difference in favor of MDT was observed in an exploratory subgroup of patients with high-grade tumors (HR 0.27, 95% CI 0.12–0.59; Holm-adjusted *p* = 0.013), representing a hypothesis-generating signal that warrants prospective validation. These findings suggest that histological grade may modify the benefit of loco-regional metastatic control in EC recurrence and should be considered in the design of future randomized trials.

## Figures and Tables

**Table 1:** Baseline characteristics of treated patients by treatment group (n = 153).

Variable	Total (n = 153)	MDT (n = 97)	Systemic (n = 56)	*p*-Value
**Age at first distant metastasis, years**				
Median (IQR)	70.0 (63.0–77.0)	69.0 (63.0–77.0)	71.0 (63.8–77.0)	0.567
**FIGO stage (2009)—n (%)**				
Stage IA	30 (19.6%)	21 (21.6%)	9 (16.1%)	0.676
Stage IB	38 (24.8%)	27 (27.8%)	11 (19.6%)	
Stage II	27 (17.6%)	15 (15.5%)	12 (21.4%)	
Stage III (not further specified)	1 (0.7%)	1 (1.0%)	0 (0.0%)	
Stage IIIA	21 (13.7%)	10 (10.3%)	11 (19.6%)	
Stage IIIB	10 (6.5%)	6 (6.2%)	4 (7.1%)	
Stage IIIC	1 (0.7%)	1 (1.0%)	0 (0.0%)	
Stage IIIC1	20 (13.1%)	13 (13.4%)	7 (12.5%)	
Stage IIIC2	5 (3.3%)	3 (3.1%)	2 (3.6%)	
**FIGO stage grouped—n (%)**				
FIGO III	58 (37.9%)	34 (35.1%)	24 (42.9%)	0.432
**Histological type—n (%)**				
Non-endometrioid	26 (17.0%)	18 (18.6%)	8 (14.3%)	0.650
**Tumor grade—n (%)**				
High grade (missing: 8)	51 (35.2%)	31 (33.7%)	20 (37.7%)	0.757
**Primary adjuvant therapy—n (%)**				
Any adjuvant therapy	111 (72.5%)	66 (68.0%)	45 (80.4%)	0.145
None	42 (27.5%)	31 (32.0%)	11 (19.6%)	
RT only	93 (60.8%)	57 (58.8%)	36 (64.3%)	
CT only	7 (4.6%)	1 (1.0%)	6 (10.7%)	
RT + CT	11 (7.2%)	8 (8.2%)	3 (5.4%)	
**Time to first distant recurrence**				
Median months (IQR)	24.0 (11.0–44.0)	24.0 (12.0–48.0)	24.0 (9.8–36.0)	0.180
0–12 months	41 (26.8%)	24 (24.7%)	17 (30.4%)	0.143
12–24 months	35 (22.9%)	24 (24.7%)	11 (19.6%)	
24–60 months	58 (37.9%)	33 (34.0%)	25 (44.6%)	
>60 months	19 (12.4%)	16 (16.5%)	3 (5.4%)	
**Number of metastatic sites at first recurrence—n (%)**				
1 site	127 (83.0%)	83 (85.6%)	44 (78.6%)	0.375
2 sites	26 (17.0%)	14 (14.4%)	12 (21.4%)	
**Metastatic site at first recurrence—n (%)**				
Pulmonary	57 (37.3%)	26 (26.8%)	31 (55.4%)	<0.001
Lymph node	18 (11.8%)	11 (11.3%)	7 (12.5%)	1.000
Peritoneal	31 (20.3%)	18 (18.6%)	13 (23.2%)	0.630
Hepatic	15 (9.8%)	4 (4.1%)	11 (19.6%)	0.005
Bone	8 (5.2%)	7 (7.2%)	1 (1.8%)	0.259
Brain	10 (6.5%)	10 (10.3%)	0 (0.0%)	0.014
Other organs	30 (19.6%)	28 (28.9%)	2 (3.6%)	<0.001
**Treatment modality—n (%)**				
Surgery alone	43 (28.1%)	43 (44.3%)	—	
RT alone	20 (13.1%)	20 (20.6%)	—	
MDT combined (surgery + RT)	15 (9.8%)	15 (15.5%)	—	
MDT + systemic	19 (12.4%)	19 (19.6%)	—	
Chemotherapy alone	40 (26.1%)	—	40 (71.4%)	
Hormone therapy alone	12 (7.8%)	—	12 (21.4%)	
Immunotherapy/targeted	3 (2.0%)	—	3 (5.4%)	
Systemic combined	1 (0.7%)	—	1 (1.8%)	

MDT = metastasis-directed therapy; IQR = interquartile range; RT = radiotherapy; CT = chemotherapy. *p*-values: Mann–Whitney U for continuous variables; χ^2^ or Fisher’s exact test for categorical variables. High grade includes FIGO grade 3, serous, clear cell, and carcinosarcoma histologies. Missing grade data: n = 8. Metastatic sites refer to the first distant recurrence (patients with two sites are counted in both). Other organs (vaginal, n = 8; soft tissue or abdominal wall, n = 8; intestinal, n = 5; urinary tract, n = 3; pelvic, n = 2; unspecified, n = 5) were all registered as distant recurrences; pleural (n = 6), skin (n = 3) and adrenal (n = 1) sites are occurred too infrequently for separate analysis and are therefore not listed as separate rows; these patients are included in the analysis cohort. Fisher’s exact test was used for bone and brain. The distribution of adjuvant modalities (none, RT only, CT only, RT + CT) differed between groups (*p* = 0.020).

**Table 2:** Cox regression analyses: overall survival from first distant metastasis.

Analysis	Covariate	HR	95% CI	*p*-Value	N
**Unadjusted analysis**					
Kaplan–Meier	MDT vs. Systemic (log-rank test)	—	—	0.202	153
**Primary analysis**					
IPTW-weighted Cox	**MDT vs. Systemic therapy**	**0.90**	0.61–1.35	0.622	145 a
**Secondary analysis—Multivariable Cox regression**					
Multivariable Cox	**MDT vs. Systemic therapy**	0.77	0.52–1.16	0.217	145
	Age at metastasis (per year)	1.03	1.00–1.05	0.026	
	FIGO III vs. I–II	0.97	0.64–1.46	0.878	
	Non-endometrioid vs Endometrioid	0.91	0.52–1.57	0.726	
	High grade vs. Low grade	2.20	1.40–3.47	0.001	
	Adjuvant therapy (Yes vs. No)	1.68	1.03–2.74	0.039	
	No. metastatic sites (2 vs. 1)	1.44	0.85–2.43	0.174	
	Early recurrence ≤ 12 m vs. >12 m	1.25	0.77–2.03	0.365	

a Excludes 8 patients with missing histological grade data. HR = hazard ratio; CI = confidence interval; MDT = metastasis-directed therapy; IPTW = inverse probability of treatment weighting with stabilized weights trimmed at the 99th percentile. Bold: primary treatment effect estimate. HR < 1 indicates improved survival with MDT.

**Table 3:** Primary analysis and key sensitivity analyses: IPTW-weighted Cox regression, overall survival from first distant metastasis.

Analysis	Description	N (MDT/Sys)	HR	95% CI	*p*-Value
Primary analysis	Strategy including MDT vs. systemic therapy alone; all treated patients	145 (92/53)	0.90	0.61–1.35	0.622
SA1	Pure MDT vs. systemic therapy (MDT plus systemic therapy excluded)	129 (76/53)	0.83	0.55–1.23	0.351
SA5	Patients with other-organ sites excluded	115 (64/51)	1.04	0.69–1.58	0.845
SA6	Time zero at treatment start (immortal-time analysis)	144 (91/53)	0.91	0.61–1.36	0.636
SA7	Patients with vaginal or pelvic lesions excluded	135 (82/53)	0.94	0.63–1.41	0.772
SA8	Patients with cerebral or other-organ metastases excluded	105 (54/51)	0.89	0.57–1.39	0.609
SA9	Calendar year of first distant metastasis added to the propensity score	145 (92/53)	0.90	0.60–1.33	0.587

All analyses used IPTW with stabilized weights trimmed at the 99th percentile and robust (sandwich) standard errors; sample sizes refer to patients contributing to each model after exclusion of the 8 patients with missing histological grade. SA2–SA4 are reported in Table S1. HR < 1 indicates improved survival with MDT. HR = hazard ratio; CI = confidence interval; MDT = metastasis-directed therapy; IPTW = inverse probability of treatment weighting; SA = sensitivity analysis.

## Data Availability

The data that support the findings of this study are held by the Tumorregister Sachsen-Anhalt and are not publicly available due to data protection regulations.

## Supplementary Materials

The following supporting information can be downloaded at: https://www.mdpi.com/article/10.3390/cancers18203382/s1, Figure S1: Love plot of covariate balance before and after inverse probability of treatment weighting; Figure S2: Sensitivity analyses, IPTW-weighted Cox regression for overall survival (SA1–SA9); Table S1: Sensitivity analyses, overall survival from first distant metastasis (SA1–SA9); Table S2: Competing risks analysis, cause-specific Cox regression results. Table S3: Exploratory subgroup analyses (Panel A) and interaction tests (Panel B) with unadjusted and multiplicity-adjusted *p*-values.
