# Supplementary material for: Metastasis-Directed Therapy Versus Systemic Therapy in Oligometastatic Endometrial Carcinoma Recurrence: A Population-Based Registry Analysis
Source: Cancers (Basel). 2026 Oct 19;18(20):3382. doi: 10.3390/cancers18203382 (PMC13702501; doi:10.3390/cancers18203382)
Supplement: Supplementary file 1 [file cancers-18-03382-s001.zip › cancers-4634559-supplementary.pdf]

Supplementary Materials

Gennari P, Busse M, Ignatov A. Metastasis-Directed Therapy versus Systemic Therapy in Oligometastatic Endometrial Carcinoma Recurrence: A Population-Based Registry Analysis.

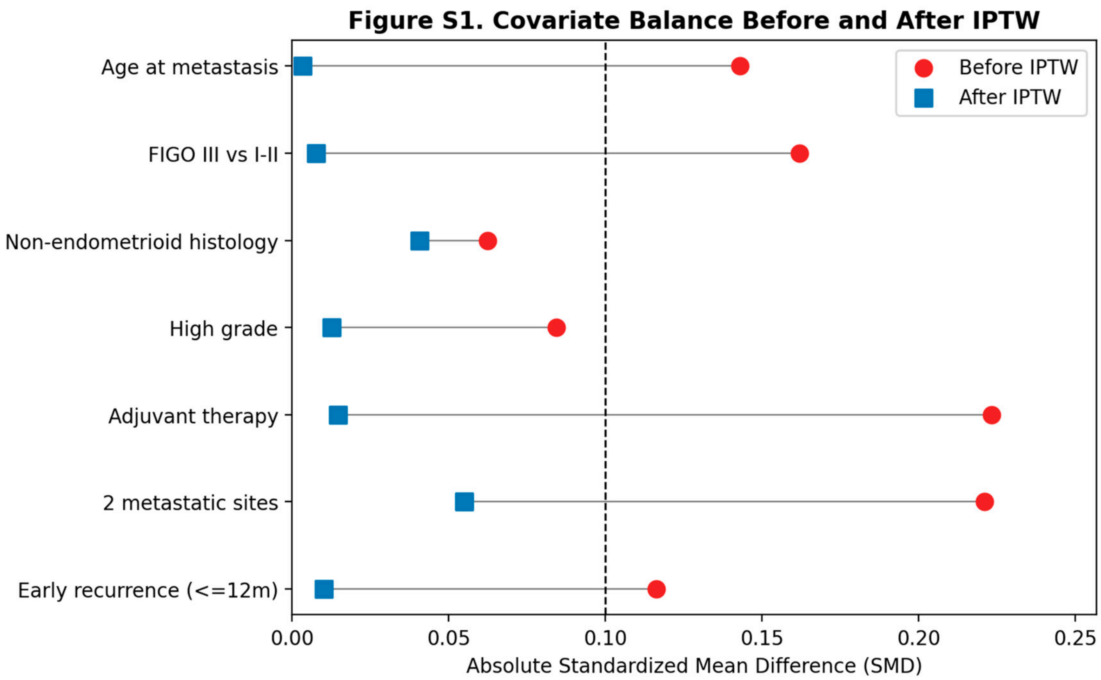

Figure S1. Love plot showing covariate balance before and after inverse probability of treatment weighting (IPTW). Red circles: absolute standardized mean differences (SMD) before IPTW. Blue squares: absolute SMD after IPTW. Dashed vertical line: SMD = 0.10 threshold for adequate balance. After IPTW, all seven covariates included in the propensity score model achieved SMD <0.10, indicating adequate balance for these covariates in the weighted sample. Metastatic site was not part of the primary propensity score model and differed between the groups at baseline (Table 1 of the main text; see SA4, SA5 and SA8 in Table S1). Propensity scores overlapped substantially between the groups (range 0.46–0.86 in the MDT group and 0.41–0.81 in the systemic therapy group); stabilized weights ranged from 0.62 to 1.62 after trimming at the 99th percentile (2 of 145 patients, 1.4%, were trimmed). IPTW = inverse probability of treatment weighting; SMD = standardized mean difference; MDT = metastasis-directed therapy; FIGO = International Federation of Gynecology and Obstetrics.

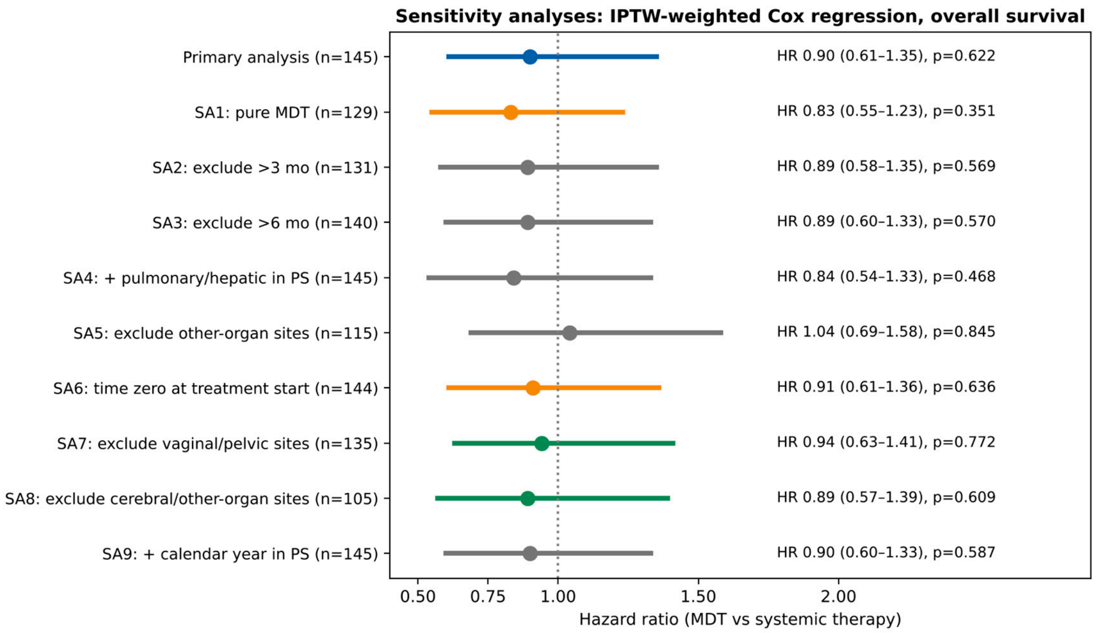

Figure S2. Sensitivity analyses: IPTW-weighted Cox regression results for overall survival (MDT vs systemic therapy). Primary analysis (blue, n=145): all treated patients classified by modality irrespective of treatment timing; MDT includes pure MDT and MDT+systemic combined. SA1 (orange, n=129): restricted to pure MDT only, excluding patients receiving concurrent systemic therapy (n=19). SA2 (grey, n=131): excludes patients whose treatment began more than 3 months after the index recurrence. SA3 (grey, n=140): excludes patients whose treatment began more than 6 months after the index recurrence. SA4 (grey, n=145): propensity score additionally adjusted for pulmonary and hepatic involvement. SA5 (grey, n=115): other-organ sites excluded. SA6 (orange, n=144): time zero at treatment start. SA7 (green, n=135): vaginal and pelvic lesions excluded. SA8 (green, n=105): cerebral and other-organ metastases excluded. SA9 (grey, n=145): calendar year of first distant metastasis added to the propensity score. All analyses used stabilized IPTW weights trimmed at the 99th percentile. HR = hazard ratio; CI = confidence interval; MDT = metastasis-directed therapy; SA = sensitivity analysis. Sample sizes refer to patients contributing to each weighted model: the restriction was applied to the 153 treated patients (SA1, 19 excluded; SA2, 14 excluded; SA3, 5 excluded; SA5, 30 excluded; SA6, 1 excluded; SA7, 10 excluded; SA8, 40 excluded) and patients with missing histological grade (n=8) were then omitted. Details of all analyses are given in Table S1.

Table S1. Sensitivity analyses: overall survival from first distant metastasis.

Analysis	Description	N (MDT/Sys)	HR	95% CI	p-value
**Primary analysis**	IPTW-weighted Cox. All treated patients classified by modality irrespective of treatment timing. MDT includes surgery alone, RT alone, MDT combined, MDT+systemic.	145 (92/53)	**0.90**	0.61–1.35	p=0.622
**SA1: Pure MDT only**	Patients receiving MDT+systemic combined (n=19) excluded. Only pure MDT (surgery, RT, or surgery+RT) vs systemic.	129 (76/53)	**0.83**	0.55–1.23	p=0.351
**SA2: exclude treatment >3 months after recurrence**	Exploratory analysis excluding patients whose treatment began more than 3 months after the index recurrence.	131 (84/47)	**0.89**	0.58–1.35	p=0.569
**SA3: exclude treatment >6 months after recurrence**	Exploratory analysis excluding patients whose treatment began more than 6 months after the index recurrence.	140 (88/52)	**0.89**	0.60–1.33	p=0.570
**SA4: additional adjustment for metastatic site**	Propensity score additionally adjusted for pulmonary and hepatic involvement at first recurrence (cerebral involvement, n=10, all MDT, could not be included because of non-overlap).	145 (92/53)	**0.84**	0.54–1.33	p=0.468
**SA5: exclude other-organ sites**	Patients with other-organ metastatic sites at first recurrence (n=30; vaginal, n=8; soft tissue or abdominal wall, n=8; intestinal, n=5; urinary tract, n=3; pelvic, n=2; unspecified, n=5; all registered as distant recurrences; 28 received MDT) excluded.	115 (64/51)	**1.04**	0.69–1.58	p=0.845
**SA6: time zero at treatment start**	IPTW-weighted Cox model with survival measured from the start of treatment instead of the first distant metastasis, removing the interval between recurrence and treatment from the observation time (post hoc; one additional patient who died before treatment start excluded).	144 (91/53)	**0.91**	0.61–1.36	p=0.636
**SA7: exclude vaginal and pelvic lesions**	Patients with vaginal (n=8) or pelvic (n=2) lesions registered as distant recurrences excluded, because such lesions may be regarded as locoregional in some settings (post hoc).	135 (82/53)	**0.94**	0.63–1.41	p=0.772
**SA8: restrict to metastatic sites present in both groups**	Patients with cerebral (n=10) or other-organ (n=30) metastases excluded, restricting the analysis to metastatic sites represented in both treatment groups (post hoc).	105 (54/51)	**0.89**	0.57–1.39	p=0.609
**SA9: adjustment for treatment era**	Propensity score additionally adjusted for the calendar year of first distant metastasis, to address changes in treatment over the 25-year study period (post hoc).	145 (92/53)	**0.90**	0.60–1.33	p=0.587

IPTW = inverse probability of treatment weighting with stabilized weights, trimmed at the 99th percentile. HR = hazard ratio; CI = confidence interval; MDT = metastasis-directed therapy; RT = radiotherapy; SA = sensitivity analysis. Covariates in the propensity score model: age at metastasis, FIGO stage, histological type, tumor grade, adjuvant therapy, number of metastatic sites, and time to recurrence. Robust (sandwich) standard errors used throughout. Sample sizes refer to patients contributing to each weighted model, i.e. after the stated restriction and after exclusion of the 8 patients with missing histological grade. SA4–SA9 were post hoc analyses. In SA9, the calendar year of first distant metastasis (continuous) was added to the propensity score model.

Table S2. Competing risks analysis — cause-specific Cox regression results.

Analysis	Event type	N	HR	95% CI	p-value
**IPTW Cox — overall survival (reference)**	All-cause death (n=115)	145	0.90	0.61–1.35	0.622
**Cause-specific Cox — primary event**	Tumor-related death (n=103)	145	0.75	0.49–1.15	0.194
**Cause-specific Cox — competing event**	Non-tumor death (n=12)	145	1.27	0.30–5.31	0.742

Cause-specific Cox regression models with MDT vs systemic therapy as the exposure variable, adjusted for age at metastasis, FIGO stage, histological type, tumor grade, adjuvant therapy, number of metastatic sites, and time to recurrence (N=145; excludes 8 patients with missing histological grade). Event of interest: tumor-related death (n=109, 71.2% of 153 treated patients; n=103 among the 145 patients in the models). Competing event: death from causes other than endometrial carcinoma (n=12, 7.8%). Censored/alive at last follow-up: n=32 (20.9%). Wide confidence interval for non-tumor death reflects the low competing event count (n=12). HR = hazard ratio; CI = confidence interval; MDT = metastasis-directed therapy; IPTW = inverse probability of treatment weighting.

Table S3. Exploratory subgroup analyses and interaction tests: unadjusted and multiplicity-adjusted p-values (Panel A: subgroup estimates).

Subgroup	Level	n	MDT/Sys	Events	HR	95% CI	p	p (Bonf.)	p (Holm)
FIGO	I-II	92	61/31	74	1.13	0.66–1.95	0.655	1.000	1.000
	III	53	31/22	41	0.41	0.20–0.85	0.016	0.196	0.180
Histology	Endometrioid	121	76/45	93	0.89	0.57–1.40	0.626	1.000	1.000
	Non-endometrioid	24	16/8	22	0.61	0.18–2.12	0.439	1.000	1.000
Grade	Low	94	61/33	66	1.29	0.76–2.20	0.339	1.000	1.000
	High	51	31/20	49	0.27	0.12–0.59	0.001	0.013	0.013
No. sites	1 site	120	79/41	94	0.70	0.45–1.11	0.131	1.000	1.000
	2 sites	25	13/12	21	0.87	0.32–2.33	0.776	1.000	1.000
Early recurrence	Late >12m	106	69/37	82	0.75	0.47–1.22	0.248	1.000	1.000
	Early <=12m	39	23/16	33	0.61	0.24–1.55	0.300	1.000	1.000
Pulmonary	Non-pulmonary	91	67/24	73	0.84	0.45–1.53	0.562	1.000	1.000
	Pulmonary	54	25/29	42	0.61	0.31–1.21	0.158	1.000	1.000

Panel B: interaction tests

Interaction (MDT × variable)	p interaction	p (Bonf.)	p (Holm)
FIGO	0.236	1.000	1.000
Histology	0.313	1.000	1.000
Grade	0.005	0.031	0.031
No. sites	0.466	1.000	1.000
Early recurrence	0.583	1.000	1.000
Pulmonary	0.402	1.000	1.000

Panel A: hazard ratios (MDT vs systemic therapy) from unweighted multivariable Cox models fitted within each subgroup (twelve estimates in total). Panel B: p-values of the MDT × subgroup-variable interaction terms (six tests) in an unweighted multivariable Cox model. p-values are unadjusted; Bonf. = Bonferroni-adjusted; Holm = Holm-adjusted for the twelve subgroup estimates (Panel A) and for the six interaction tests (Panel B), respectively. Subgroup analyses were exploratory and are hypothesis-generating. Sample sizes refer to the 145 patients with complete data. HR = hazard ratio; CI = confidence interval; MDT = metastasis-directed therapy; Sys = systemic therapy.
